# Relevance of the protein macrodipole in the membrane-binding process. Interactions of fatty-acid binding proteins with cationic lipid membranes

**DOI:** 10.1371/journal.pone.0194154

**Published:** 2018-03-08

**Authors:** Vanesa V. Galassi, Marcos A. Villarreal, Guillermo G. Montich

**Affiliations:** 1 Universidad Nacional de Córdoba, Facultad de Ciencias Químicas, Departamento de Química Biológica “Ranwel Caputto”, Córdoba, Argentina; 2 CONICET, Universidad Nacional de Córdoba, Centro de Investigaciones en Química Biológica de Córdoba (CIQUIBIC), Córdoba, Argentina; 3 Universidad Nacional de Córdoba, Facultad de Ciencias Químicas, Departamento de Química Teórica y Computacional, Córdoba, Argentina; 4 CONICET, Universidad Nacional de Córdoba. Instituto de Investigaciones en Fisicoquímica de Córdoba (INFIQC), Córdoba, Argentina; University of Waterloo, CANADA

## Abstract

The fatty acid-binding proteins L-BABP and Rep1-NCXSQ bind to anionic lipid membranes by electrostatic interactions. According to Molecular Dynamics (MD) simulations, the interaction of the protein macrodipole with the membrane electric field is a driving force for protein binding and orientation in the interface. To further explore this hypothesis, we studied the interactions of these proteins with cationic lipid membranes. As in the case of anionic lipid membranes, we found that both proteins, carrying a negative as well as a positive net charge, were bound to the positively charged membrane. Their major axis, those connecting the bottom of the β-barrel with the α-helix portal domain, were rotated about 180 degrees as compared with their orientations in the anionic lipid membranes. Fourier transform infrared (FTIR) spectroscopy of the proteins showed that the positively charged membranes were also able to induce conformational changes with a reduction of the β-strand proportion and an increase in α-helix secondary structure. Fatty acid-binding proteins (FABPs) are involved in several cell processes, such as maintaining lipid homeostasis in cells. They transport hydrophobic molecules in aqueous medium and deliver them into lipid membranes. Therefore, the interfacial orientation and conformation, both shown herein to be electrostatically determined, have a strong correlation with the specific mechanism by which each particular FABP exerts its biological function.

## Introduction

Electrostatic interactions are recognized as major contributing forces for membrane binding of peripheral membrane proteins [1–5]. The functionality, or biological activity of these proteins is strongly defined by the orientation within the membrane anisotropic environment and this orientation is -to a large extent- also defined by electrostatic interactions.

We attempted to prove the relevance of electrostatic interactions in defining the orientation of peripheral membrane proteins. We have previously studied the binding to lipid membranes of the chicken liver bile acid-binding protein [6], (L-BABP, PDB ID 1TVQ [7]) and the regulatory protein of the squid nerve sodium calcium exchanger [8], (ReP1-NCXSQ, PDB 3PPT [9]). They share the common fold of the family: a β-barrel delimiting an inner cavity and a portal region with two α-helix segments. We demonstrated, using binding assays and Molecular Dynamics (MD) simulations, that they interact with negatively charged lipid membranes and that electrostatic interactions have a major role on determining their orientation in the interface [6, 8, 10–13]. We have shown that the alignment of the dipole moment with the membrane electric field has a dominant role in the protein orientation within the interface.

ReP1-NCXSQ binds to anionic lipid membranes even when it is negatively charged in solution at neutral pH. Using Poisson Boltzman mean field theory in a rigid protein representation and a continuum model for the membrane, Zamarreño et al. [14] have also shown the influence of dipole-field interactions on the orientation of the protein and on the strength of binding. Those works strongly suggest that the interaction between the macrodipole of the proteins and the membrane electric field contributes not only to orientation but also to the binding to the interface. Then, a positively charged interface should also bind proteins with large macrodipoles in an opposite orientation and, to some extent, irrespective of the macromolecule net charge. Mulgrew-Nesbitt et al. [5] have reviewed the relevance of the charge distribution rather than the net charge. Here we put this hypothesis under a severe test: if the electrostatic binding and orientation of FABPs to lipid membranes is influenced by the dipole-field interaction, it should also work for cationic lipid membranes. Here we studied the interactions of L-BABP and ReP1-NCXSQ with cationic lipid membranes experimentally and by MD simulations.

Cationic lipids are minor components of biological membranes and hardly participate in unspecific long-range interactions determined by the membrane electric fields. Hence, our system was not designed to mimic a particular interaction expected to be found in the physiology of the living cell. Instead, we used the cationic lipid membranes to prove that the asymmetric distribution of charges in proteins is a determining factor for the orientation at charged membrane interfaces.

Our present results may have a practical consequence related to the cell transfection and drug delivery systems. Cationic lipid liposomes are currently used to deliver genetic material for transfection [15], drugs [16] and interference RNA [17] into target cells. They have proven to be more efficient than anionic liposomes to bind the target and deliver the cargo, but they are also more toxic and more prone to bind circulating and membrane peripheral proteins [16, 18]. It was shown that cationic liposomes recruit and bind preferentially acidic proteins from serum plasma through an obvious interaction between opposite electric charges [19]. Still, it is worth considering that positively charged proteins can also be bound through a field-dipole interaction as we demonstrate in this work.

Several works have addresed the computer simulation of systems containing cationic lipids with the interest of undestanding the interactions with nucleic acids and drugs that can potentially be transported. As a result of these studies, new knowledgement was acquired about the organization, phase behaviour, segregation of lipid components and properties of the electric double layer in cationic interfaces [20–24].

## Materials and methods

Purified L-BABP was kindly supplied by Dr. Hugo Monaco and stored in 2 mM phosphate buffer, pH 7.5, at -70 ^o^C. Recombinant ReP1-NCXSQ was expressed and purified as in Galassi et al. [8].

Lipids were from Avanti Polar Lipids (Alabaster, AL). Nucleoeléctrica Argentina S.A. Central Nuclear Embalse, Div. Química y Procesos supplied ^2^H_2_O 99.9+%. Microcon YM100 concentrators were from Amicon (Beverly, MA).

### Large unilamellar vesicles preparation

Large unilamellar vesicles (LUVs) were prepared by extrusion through polycarbonate filters with pores of 100 nm diameter as in Nolan et al. [10]. Dynamic light scattering showed that the vesicles were 60 nm diameter for 1,2-dimyristoyl-3-trimethylammonium-propane (DMTAP) and 1,2-dimyristoyl-sn-glycero-3-ethylphosphocholine (EDMPC) and 90 nm for 1,2-dimyristoyl-sn-glycero-3-phosphoglycerol (DMPG). For the FTIR experiments, we prepared the LUVs in ^2^H_2_O.

### Filtration assays

Mixtures of LUVs and proteins were incubated for 30 min at 33 ^o^C, loaded in the upper chamber of YM100 concentrators and spun down at 7500 × g until 80% of the initial volume was eluted. Aqueous solutions contained 10, 100 and 500 mM NaCl and 3 mM phosphate buffer. The final pH was 6.8. The initial mixtures contained 5 μM protein and 500 μM lipid. YM100 filters allow the elution of the unbound protein and retain the lipid vesicles together with membrane-bound protein. The protein concentration in the initial sample and in the eluted fraction was quantified by the absorbance at 280 nm. The concentration of eluted protein in the absence of LUVs was taken as 100% of elution.

### Molecular dynamics simulations

A symmetric bilayer composed of 128 molecules of EDMPC was assembled, hydrated (~3500 water molecules), neutralized with Cl^-^ ions, pre-equilibrated (200 ns) and placed in a 7 × 7 × 10 nm^3^ triclinic box containing a protein molecule, neutralizing Na^+^ and Cl^-^ ions and ~10^4^ water molecules. Four different initial configurations for ReP1-NCXSQ and two for L-BABP were generated by placing the proteins in different orientations and distances with respect to the interfacial plane. The distance between the protein center of mass (COM) and the *cis* hemilayer plane (determined by the phosphorous atoms average position) was between 2.5 and 4.5 nm, which is about the thickness of 7 to 13 layers of water.

The GROMOS96 force field [25] was used with the G53A6 set of parameters [26] for the protein. The lipid parameters were from Kukol [27] for 1,2-dimyristoyl-sn-glycero-3-phosphocholine (DMPC) modified by the addition of an ethyl group to the phosphate group. Kukol's atomic types, and also the charges for the glycerol group and the hydrocarbon chains were conserved, while an *ab initio* calculation for the headgroup remaining charges was performed using a PM3 optimized geometry. MOPAC partial charges were assigned using ATB webserver [28]. The charges and atomic types for the lipid molecule are specified in the Supporting Information (S1 Fig, S1 Table). The simple point charge (SPC) model of water was used [29]. The charges on the aminoacid side chains were according to the pKa estimated by the PROPKA web server [30]. The electrostatic interactions were handled with the SPME version of the Ewald sums [31], with a real space cutoff of 0.9 nm. The van der Waals interactions were handled with the twin-range scheme with the short range cutoff at 0.9 nm and the long range cutoff at 1.4 nm. The simulations were carried out in the NPT ensemble using the v-rescale thermostat [32] and Berendsen barostat [33]. The protein, the membrane, and the solvent were coupled separately to a temperature bath with a reference temperature of 320 K and a relaxation constant of 0.2 ps. The pressure was maintained constant by coupling to a reference pressure of 1 bar with a relaxation constant of 2.0 ps and a compressibility of 5×10^−5^ bar^-1^. Semi-isotropic coupling in the direction normal to the bilayer was applied. No dispersion corrections were applied neither in long range interactions nor in the pressure, to avoid artifacts in the mean area per lipid and in the bilayer thickness [34]. The bonds in the protein and the lipids were constrained using the LINCS algorithm [35]. For the bonds and angle of the water molecules, we used the SETTLE algorithm [36]. The time step for the integration of the equation of motion was 5 fs due to the use of virtual sites in the polar hydrogen atoms of the protein and the lipids [37]. The non-bonded list was updated every 4 time steps. Every run, whether of equilibration or production, was started with a different set of initial velocities in order to produce non-correlated trajectories. Production runs were performed without restrictions during 100 ns. The simulations and their analysis were performed with the GROMACS 4.5.4 software package [38].

Macrodipoles were computed using the GROMOS96 partial atomic charges localized according to the crystallographic structures. The electrostatic equipotential contour calculations were performed through the Finite Difference Poisson Boltzmann Equation method as implemented in APBS (Adaptive Poisson Boltzmann Solver) [39] for VMD software package [40].

### FTIR spectroscopy

The protein was lyophilized from an aqueous solution, dissolved in 10 mM NaCl, 3 mM phosphate buffer in ^2^H_2_O and incubated overnight at room temperature. Mixtures of LUVs and proteins were incubated at 33 ^o^C for 30 min and then transferred to a thermostated cell for liquid samples with CaF_2_ windows and 75 μm spacers. Spectra were recorded in a Nicolet Nexus spectrometer. Fifty scans were collected for the background and the sample at 2 cm^-1^ resolution. A linear baseline was defined between 1600 and 1700 cm^-1^ and the area was normalized to unity between these limits. Component bands of the measured spectra were identified by Fourier self-deconvolution (FSD) and second derivative. Band fitting was performed according to Arrondo et al. [41] and Nolan et al. [10].

## Results

### Electrostatic properties of L-BABP and ReP1-NCXSQ: protein macrodipole and net charge

**Fig 1:**
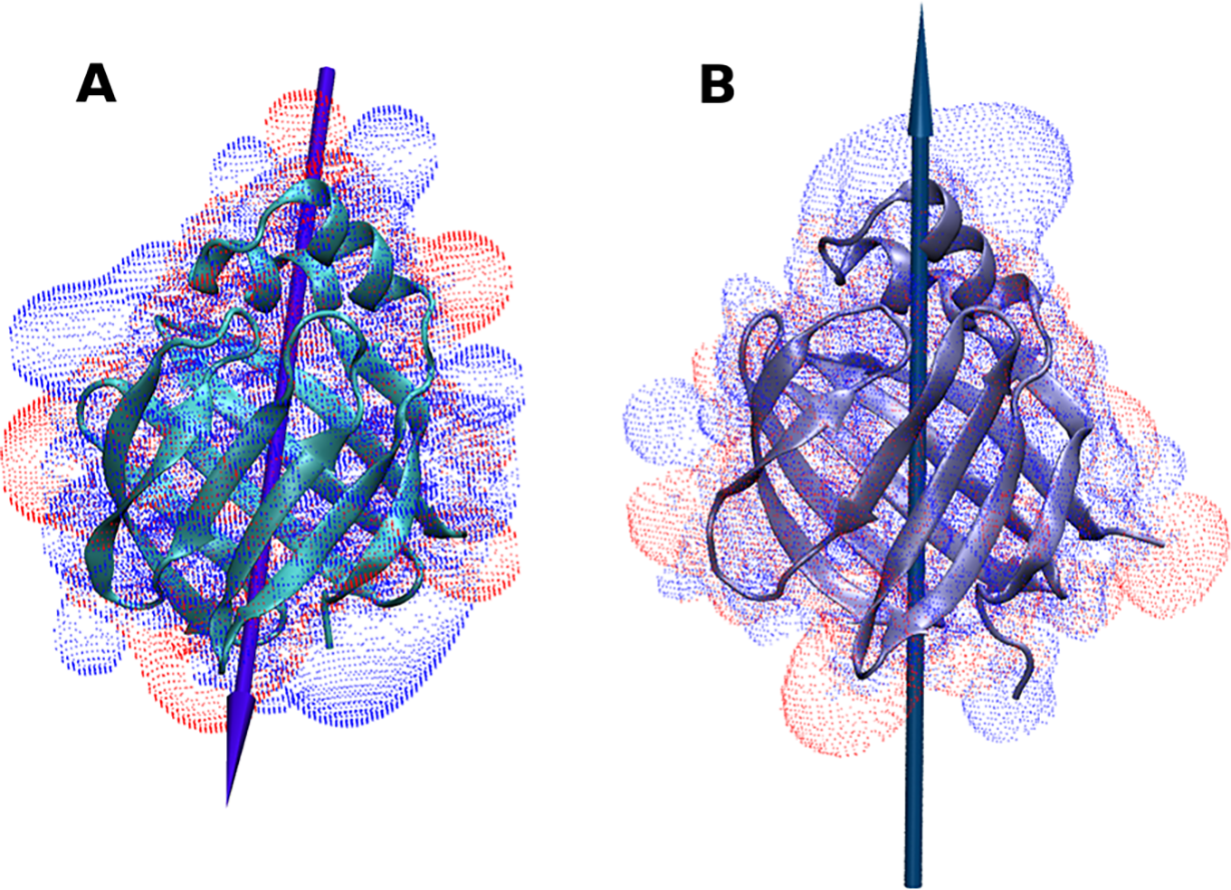
Charge distribution of ReP-NCXSQ and L-BABP. Cartoon representation of L-BABP (panel A) and ReP1-NCXSQ (panel B). Macrodipoles are represented as arrows. The macrodipole vector of L-BABP is scaled 2.5 times greater than ReP1-NCXSQ to facilitate visualization. The electrostatic potential is represented in transparent surface; the isosurfaces were set in +1 mV (blue) and -1 mV (red).

Both proteins, L-BABP and ReP1-NCXSQ, have a net electric charge in solution at neutral pH. L-BABP has an isoelectric point pH(I) = 9, and a net charge z = +2, while the isoelectric point of ReP1-NCXSQ is pH(I) = 5.85, with a charge z = -1 at pH 7. The macrodipoles and the isopotential surfaces at an electrostatic potential of -1 mV in L-BABP are shown in Fig 1. Visual inspection of the charge distribution did not reveal an obvious charge separation. ReP1-NCXSQ displayed a positive lobule that matched the positive end of the macrodipole, but no negative pole could be identified. In L-BABP, a net charge separation was less evident. The macrodipole calculation, instead, produced a clearer picture of the charge asymmetry. Using the approach by Porschke [42], we calculated that the macrodipole of L-BABP is 175 D, oriented along the barrel, with the positive end pointing towards the base of the barrel, and 400 D for ReP1-NCXSQ with the positive end pointing towards the portal region. The protein dipole arises from the spatial arrangement of both the backbone and the aminoacid lateral groups. In β-barrels in particular, the axial symmetry of the folding determines that the largest contribution to the macrodipole is given by the lateral chains of the aminoacids, specially from the charged ones [6].

### Binding to cationic lipid membranes

Two different cationic lipids were used: DMTAP and EDMPC. It is relevant to take into account that the electrostatic surface potential of analogs of DMTAP (1,2-dioleoyl-3-trimethylammonium-propane, DOTAP) and of EDMPC (1,2-dipalmitoyl-sn-glycero-3-ethylphosphocholine, EDPPC) at neutral pH are +200 and +150 mV respectively [43, 44]. We performed binding assays in which the unbound protein was eluted and quantified. Table 1 shows the percentages of eluted protein in filtration binding assay for the different systems. Both proteins were bound to the cationic lipid membranes at a bulk pH = 6.8 and low ionic strength (10 mM NaCl) although a larger proportion of ReP1-NCXSQ was bound as compared to L-BABP. This correlates with a lower macrodipole and the positive net charge in L-BABP.

**Table 1:** Percentage of eluted protein from filtration assays.

		L-BABP		ReP1-NCXSQ	
		DMTAP	EDMPC	DMTAP	EDMPC
pH 6.8	10 mM NaCl	18.6	21.2	8.0	6.4
	100 mM NaCl	65.1	83.7	40.3	46.8
	500 mM NaCl	83.7	93.0	56.4	79.0
pH 5.0	10 mM NaCl	97.3	89.2	2.5	10.0

A brief discussion about the net charge of a protein at the interface is relevant at this point. The effective pKa of molecules located in charged interfaces can be up to one unit different as compared to the same group in bulk solution. Even when the activity of the H^+^ ion is constant along the system [45], the effect can be rationalized considering that the interfacial pH is lower in the anionic lipid membranes and higher in the positively charged interface. It is difficult to take into account this effect to evaluate the exact protonation state in the interface because the pH changes occur within distances comparable to the size of the proteins; some residues can be exposed to the bulk solution while others to the interfacial pH. To evaluate the protonation state of the proteins, we considered the pH of the bulk solution. For both proteins, the net charge remained with the same sign within the pH range 5 to 9, according to the pKa values estimated by PROPKA [30]. Consequently, our conclusion regarding the relevance of the net charge is still valid even if we consider the variations of the interfacial pH.

No difference was observed in the extent of binding between DMTAP and EDMPC, suggesting that there were no specific interactions and that the binding was mainly electrostatic. Increasing NaCl concentration to 100 and 500 mM produced a decrease in the amount of membrane-bound protein. We have also measured the binding at different pHs (see Table 1). Decreasing the bulk pH to 5.0 produced a decrease in the association of L-BABP to cationic membranes. At this pH, three glutamates (Glu25, Glu94 and Glu101) and two histidines (His83 and His98) were protonated according to the pKa estimated using the Poisson Boltzman model [30] and considering the bulk pH. We evaluated that the net charge of the protein increased to z = +7 and the macrodipole decreased to 110 D. We attributed the decreased binding to the electrostatic repulsion of the increased net charge with the positively charged membrane, which outbalanced the weakened dipolar interaction. Conversely, the association of ReP1-NCXSQ was not significantly affected by the change in pH. Only two residues were predicted to be protonated in this protein: Glu72 and His113, conferring a net charge of z = +1. The dipole decreased to 360 D, which is a much smaller change than in L-BABP.

These proteins were not bound to zwitterionic lipid membranes and their binding to the charged membranes was dependent on the ionic strength. These observations strongly suggest that the binding to the cationic membrane was electrostatic. These results can be explained if we consider that the interactions of both the net electric charge and the macrodipole with the membrane electric field determine the strength of binding. As concluded for the case of L-BABP at neutral pH, the repulsion between the positive interface and the positive charge z = +2 is outbalanced by the interaction of the macrodipole of 175 D with the interfacial electric field. In an acidic medium, when the charge was increased and the dipole was decreased, the contribution of the dipolar interaction was not strong enough to drive the binding against the repulsion of the net charge.

### Orientation in cationic lipid membranes

**Fig 2:**
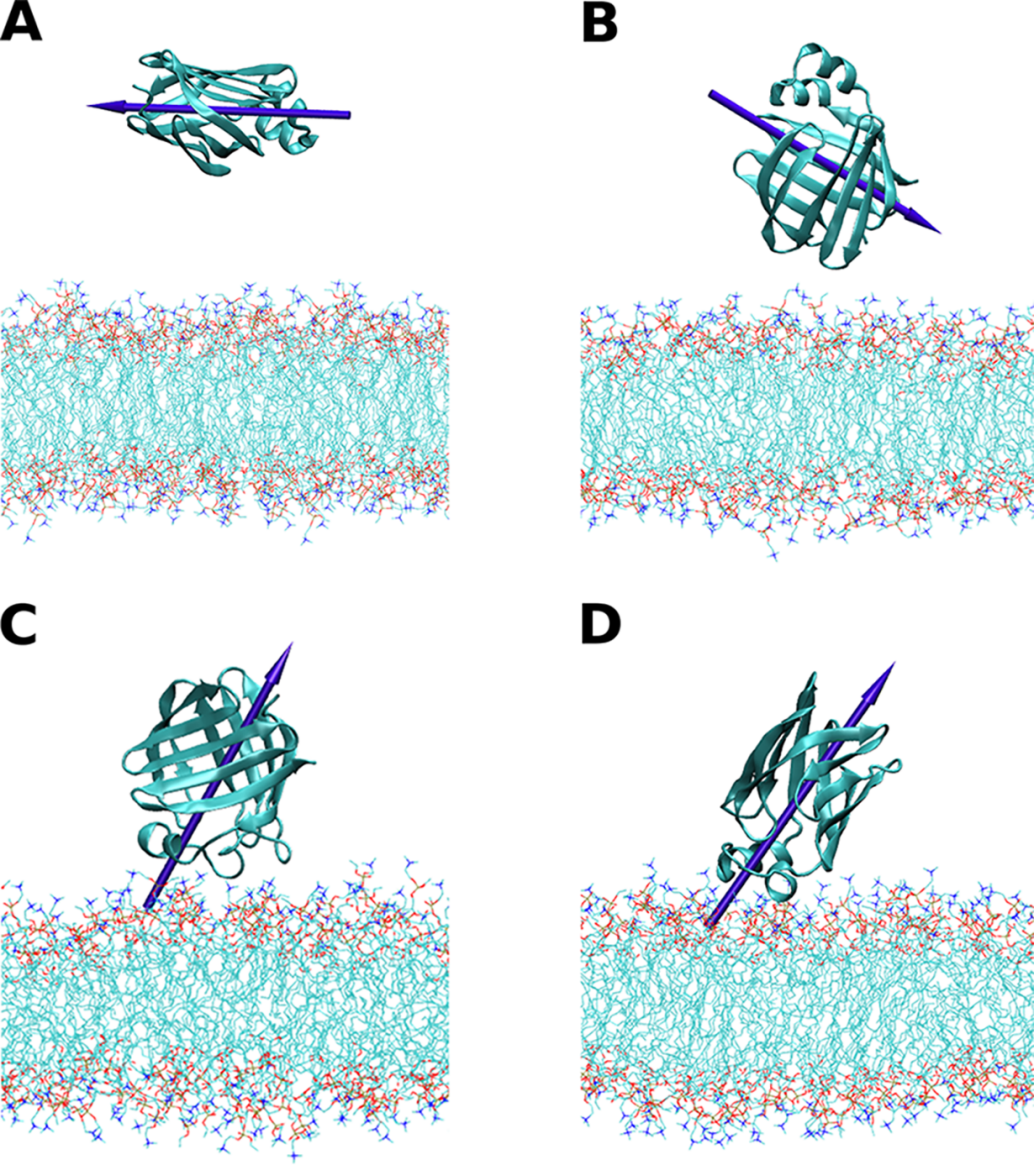
Initial and final configurations of the simulations of L-BABP. Initial (upper panels) and final (lower panels) configurations of the 100 ns simulations of L-BABP with EDMPC at pH 6.8, 10 mM NaCl. B-EDMPC1 (panels A and C) and B-EDMPC2 (panels B and D). The arrows represent the macrodipoles. N atoms in choline and O atoms in phosphate groups are in blue and red respectively.

**Fig 3:**
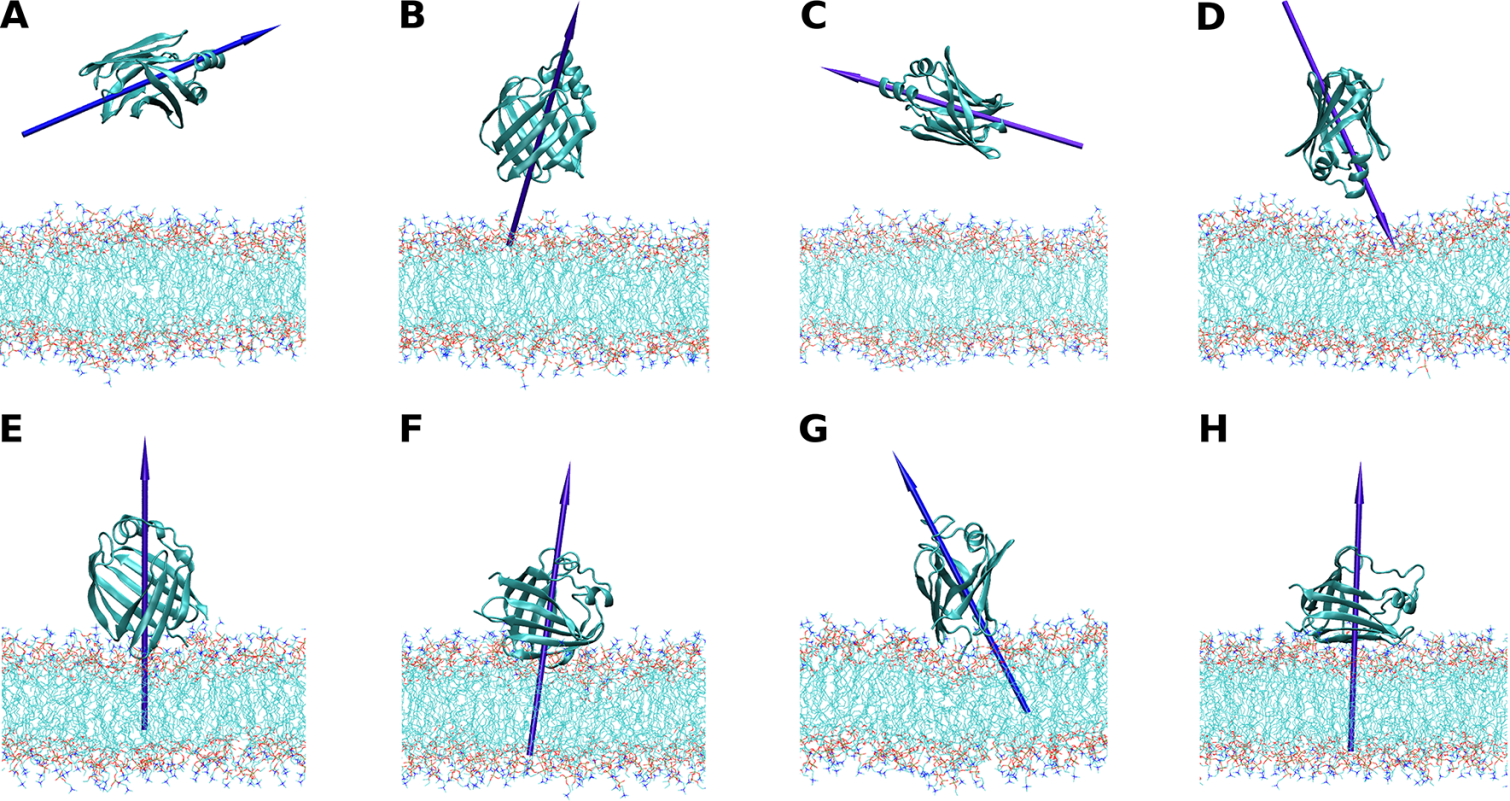
Initial and final configurations of the simulations of ReP1-NCXSQ. Initial (upper panels) and final (lower panels) configurations of ReP1-NCXSQ in EDMPC at pH = 6.8, 10 mM NaCl. R-EDMPC1 (panels A and E), R-EDMPC2 (panels B and F), R-EDMPC3 (panels C and G) and R-EDMPC4 (panels D and H). The arrows represent the macrodipoles. N atoms in choline and O atoms in phosphate groups are in blue and red respectively.

We performed simulations of 100 ns to study the binding of L-BABP and ReP1-NCXSQ to the cationic bilayer of EDMPC. Simulations started with L-BABP and ReP1-NCXSQ in the aqueous phase in a simulation box containing the lipid membrane. The reaction coordinate was the distance between the protein COM and the membrane surface plane in the *cis* leaflet (*z*_*L-P*_). We made simulations with several initial configurations, four for ReP1-NCXSQ and two for L-BABP, with different orientations and with *z*_*L-P*_ ranging from 2.5 to 4.5 nm. Fig 2 and Fig 3 show the initial and final configurations.

**Fig 4:**
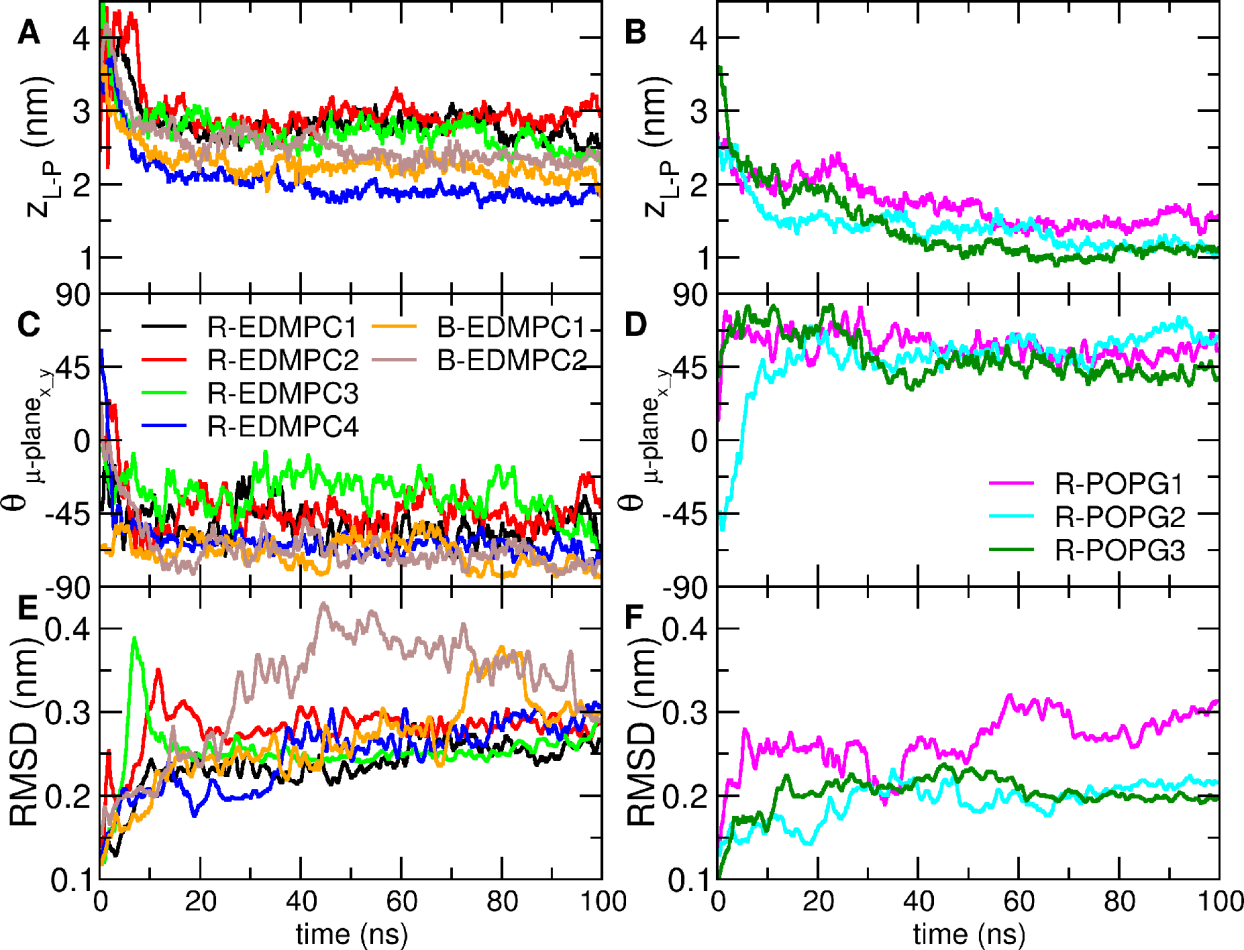
Trajectories of the molecular dynamics simulations of adsorption. The *z*_*L-P*_ (panel A and B), the *θ*_*μ-plane x-y*_ (panel C and D) and the root mean square deviation (RMSD) (panel E and F) for the four simulations of ReP1-NCXSQ with cationic membranes of EDMPC (R-EDMPC1-4) and the two of L-BABP with EDMPC (B-EDMPC1-2) in panels A, C and E. In order to have a reference, data from simulations of ReP1-NCXSQ with anionic membranes of POPG (R-POPG1-3) [8] are displayed in panels B, D and F.

**Fig 5:**
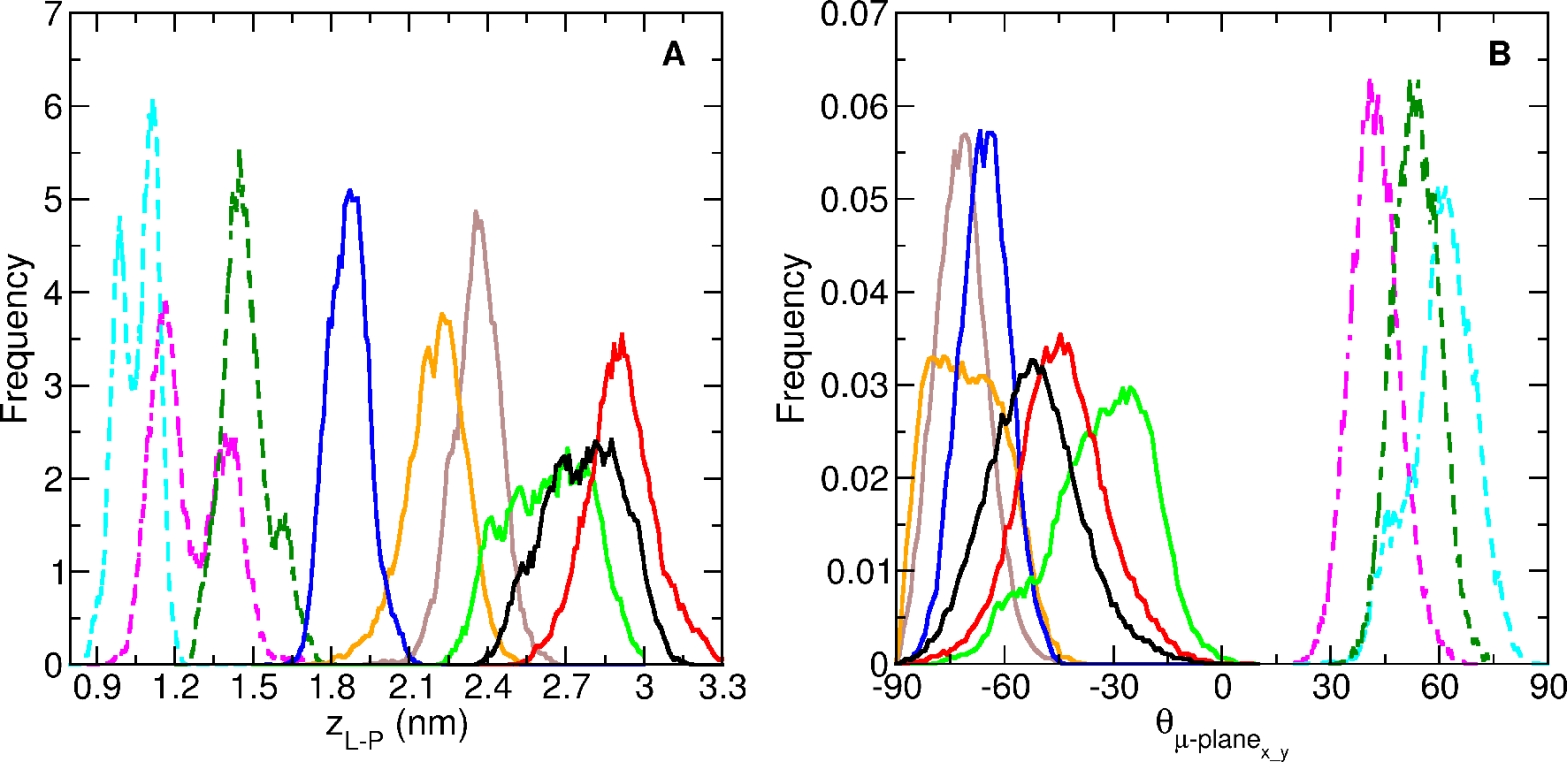
Histograms of the “bound” state over the last 70 ns of the molecular dynamics simulations of the adsorption processes. *z*_*L-P*_ (panel A) and *θ*_*μ-plane x-y*_ (panel B) for the four simulations of ReP1-NCXSQ with cationic membranes of EDMPC, the two of L-BABP with EDMPC, and of ReP1-NCXSQ with anionic membranes of POPG [8]. Colour coding are as in Fig 4.

Fig 4A shows the trajectories of *z*_*L-P*_ for ReP1-NCXSQ and L-BABP in the presence of EDMPC cationic membranes. For comparison, we also show in Fig 4B the same property computed for ReP1-NCXSQ in anionic membranes [8]. Both proteins migrated to the membrane and reached a constant distance within 1.75 and 2.75 nm. The average distances were larger than the observed in anionic membranes (see the histograms of the “bound” state (last 70 ns of the simulations) in Fig 5). A remarkable result was that even when the initial orientations were different for all simulations, the proteins acquired single well-defined orientations: the portal domain in L-BABP and the base of the β-barrel in ReP1-NCXSQ were oriented towards the membrane at the end of simulations. The orientation of the macrodipoles, and consequently of the proteins, were rotated 180^o^ as compared with the orientations previously observed, for each protein, in anionic lipid membranes [6, 8].

ReP1-NXSQ was oriented with angles *θ*_*μ-plane x-y*_ between -75^o^ and -60^o^ (except for R EDMPC3 where the mean *θ*_*μ-plane x-y*_ angle was -30^o^), and L-BABP adopted *θ*_*μ-plane x-y*_ values between -55^o^ and -50^o^ (Figs 4B, 4C and 5B). As we previously discussed [8], we attribute this biased orientation to the contribution of the lowest energy configuration of the macrodipole in the interfacial electric field. In all the simulations it was observed that the macrodipole was aligned parallel to the normal to the membrane, even when it required a rotation of 180^o^, as for example in R-EDMPC4. In the case of R-EDMPC3, we attribute the lower alignment to a lack of convergence: it can be noted that during the last 10 ns of simulation the dipole rotated towards a parallel orientation. In all cases, these orientations were opposite to that observed in anionic membranes, where the macrodipole alignment follows the electric field generated by the negatively charged lipid interface [8].

### ReP1-NCXSQ conformational changes in cationic lipid membranes

**Fig 6:**
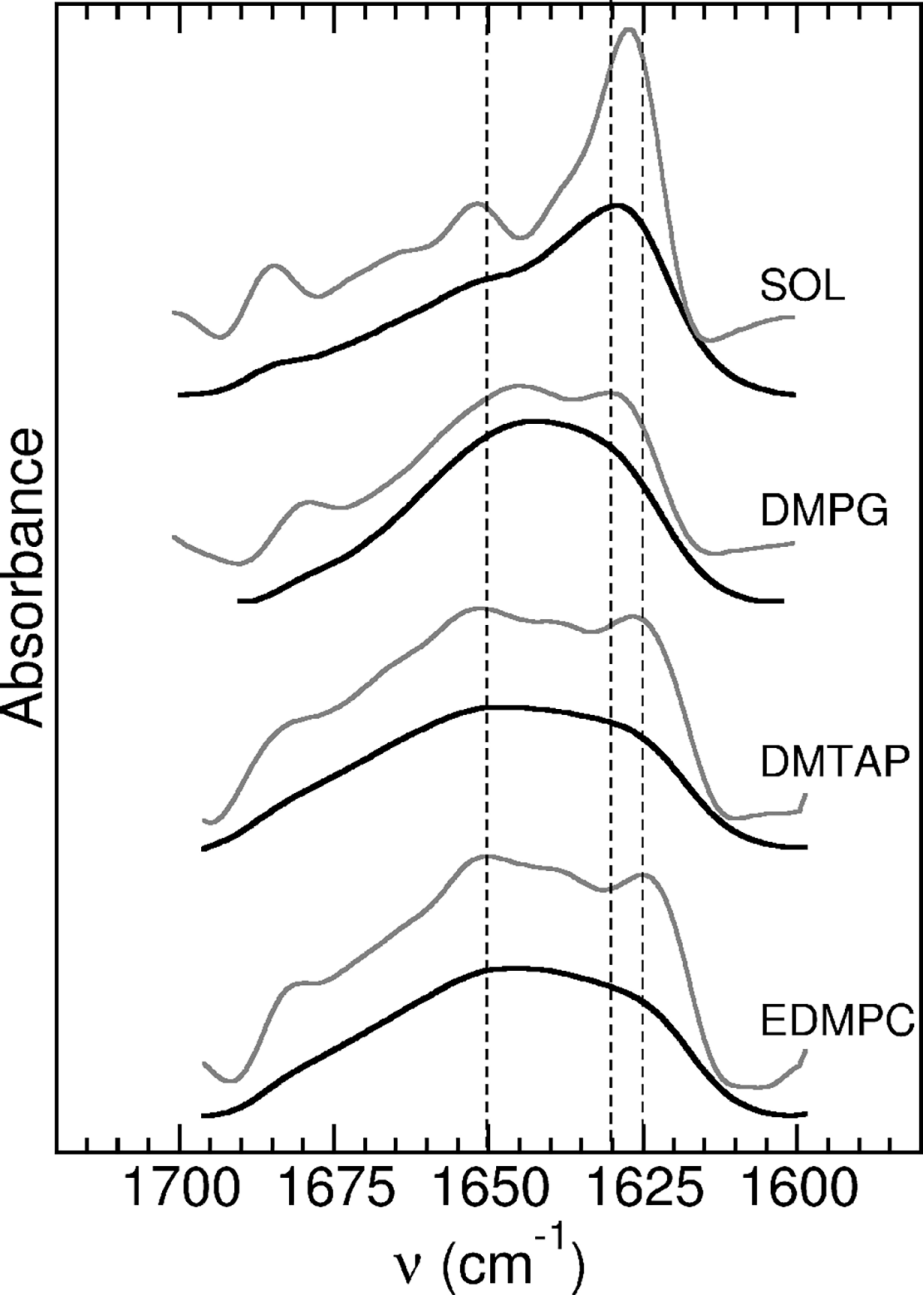
FTIR spectra of ReP1-NCXSQ. Spectra in solution (SOL) and in the presence of anionic membranes of DMPG and cationic membranes of DMTAP and EDMPC in this order from top to bottom. Samples were at 33 ^o^C. Measured spectra: lower black traces. Fourier self-deconvolutions: upper gray traces, band width = 18 cm^-1^ and factor k = 2. For reference, dotted lines were traced at 1625, 1630 and 1650 cm^-1^.

**Fig 7:**
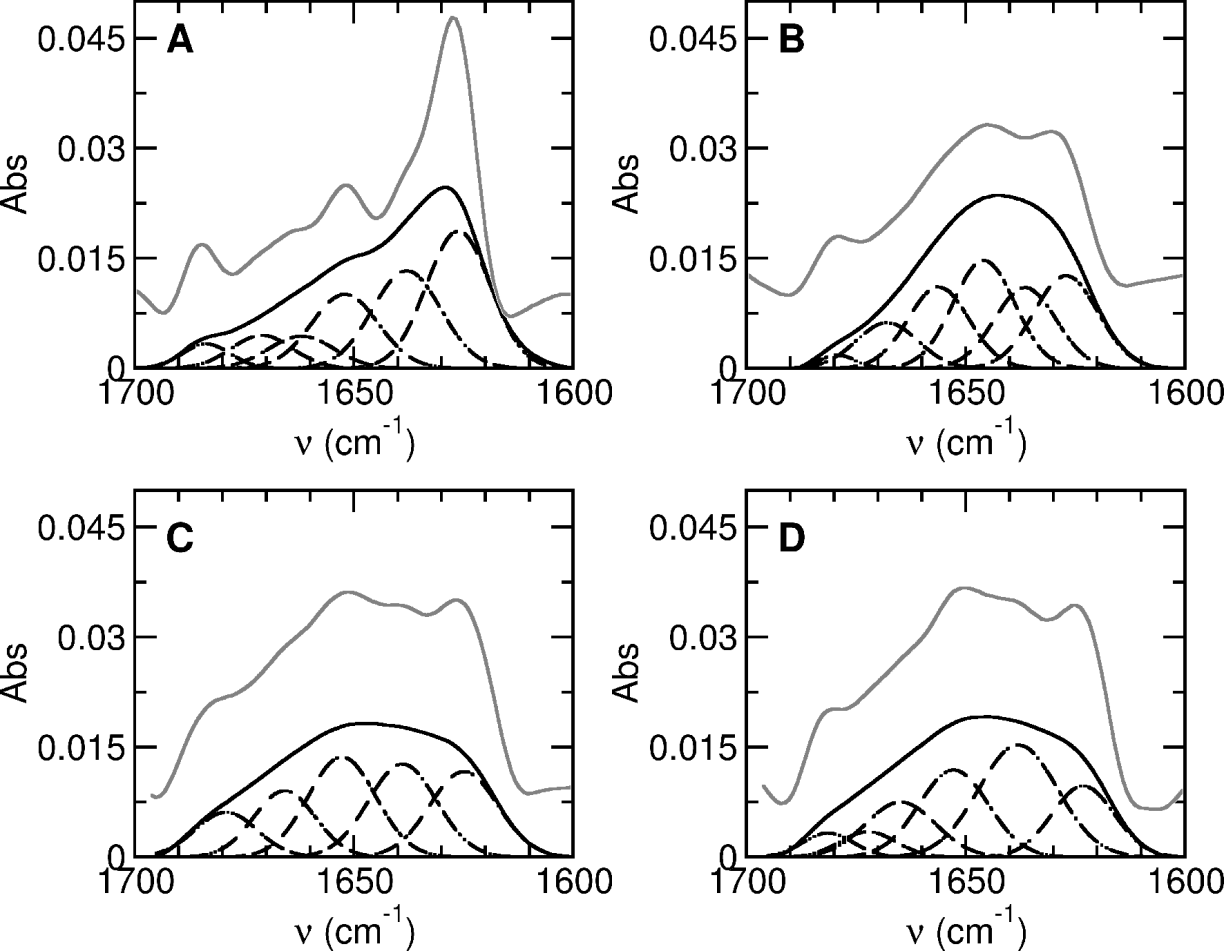
Component analysis of the FTIR spectra. ReP1-NCXSQ in solution (panel A) and in the presence of anionic membranes of DMPG (panel B) and cationic membranes of DMTAP (panel C) and EDMPC (panel D). Spectra were collected a 33 ^o^C. Measured spectra (lower continuous black trace), Fourier self-deconvolutions (upper continuous gray trace), using bandwidth of 18 cm^-1^ and factor k = 2. Band components are in dashed lines.

We have also studied the ability of cationic lipid membranes to induce conformational changes, as already observed in anionic lipid membranes for L-BABP [6, 10, 11, 13, 46] and for ReP1-CXSQ [8]. We measured the infrared spectra of ReP1-NCXSQ in solution and in the presence of LUVs of cationic membranes of DMPTAP and EDMPC, and anionic membranes of DMPG at 33 ^o^C (Fig 6). In solution, at 33 ^o^C, ReP1-NCXSQ showed a FTIR spectrum typical of a protein with a large amount of β-secondary structure. The bands at 1626, 1638 and 1671 cm^-1^ are assigned to β-strands and contributed with 68% to the total area (Fig 7). The band assigned to α-helix at 1652 cm^-1^ accounted for 19% of the area. The bands at 1662 and 1684 cm^-1^, contributed to 13% of the total area. This is in agreement with previous results [47]. The FTIR spectrum remained unchanged when the protein was mixed with zwitterionic membranes (not shown). Instead, when it was mixed with the charged membranes we observed large spectral changes. In all cases, the band at 1626 cm^-1^ decreased and it was slightly shifted (see Table 2). In the protein bound to DMPG the second derivative of the FSD spectrum revealed a band at 1645 cm^-1^ due to unordered structure. The band assigned to helical structure was increased (see Table 2). The binding to cationic membranes also produced remarkable spectral changes, to some extent similar to the observed in the anionic membrane. The band assigned to unordered structure was not present. The large spectral changes in the presence of charged membranes also evidenced that ReP1-NCXSQ interacted both with anionic and cationic interfaces. The different conformations acquired in anionic as compared with cationic membranes can be due to different degrees of hydration, hydrogen bonds network, and electric field strength, among others. An interesting possibility is that they were determined by the domain of the protein that interacted with the membrane, whether it was the base of the barrel in the cationic interface, or the helix domain in the anionic membranes as observed in the simulations [8].

**Table 2:** Components of the amide I band of ReP1-NCXSQ and proportion of secondary structure determined by band fitting.

	Solution		DMPG		DMTAP		EDMPC	
	Band position, cm^-1^	% area	Band position, cm^-1^	% area	Band position, cm^-1^	% area	Band position, cm^-1^	% area
β structure	1626	34	1624	19	1625	21	1625	17
β structure	1638	26	1635	27	1639	27	1637	34
unordered			1645	16				
α-helix	1652	19	1654	23	1653	25	1651	24
Turns	1662	8	1667	13	1666	15	1666	14
β structure	1671	8					1671	5
Turns	1684	5	1680	2	1680	11	1680	6

We computed the root mean square deviation (RMSD) from a reference conformation for both proteins along the simulations (see Fig 4E and 4F). The references were the most sampled conformation in solution and they were deviated only 0.1 nm from the crystallographic structure. The RMSD increased 0.22–0.30 nm for proteins, similar to the values observed in anionic membranes [6, 8]. No major changes in secondary structure were detected by DSSP analysis [48] (S2 Fig). Conformational changes require concerted changes in torsion angles and occur within a timescale in the order of μs. These changes are not likely to occur within the time span of our simulation, but the increase in the RMSD values when proteins were bound to the membrane evidences fluctuations that may drive conformational changes at longer simulation times (S2 Fig).

## Discussion

**Fig 8:**
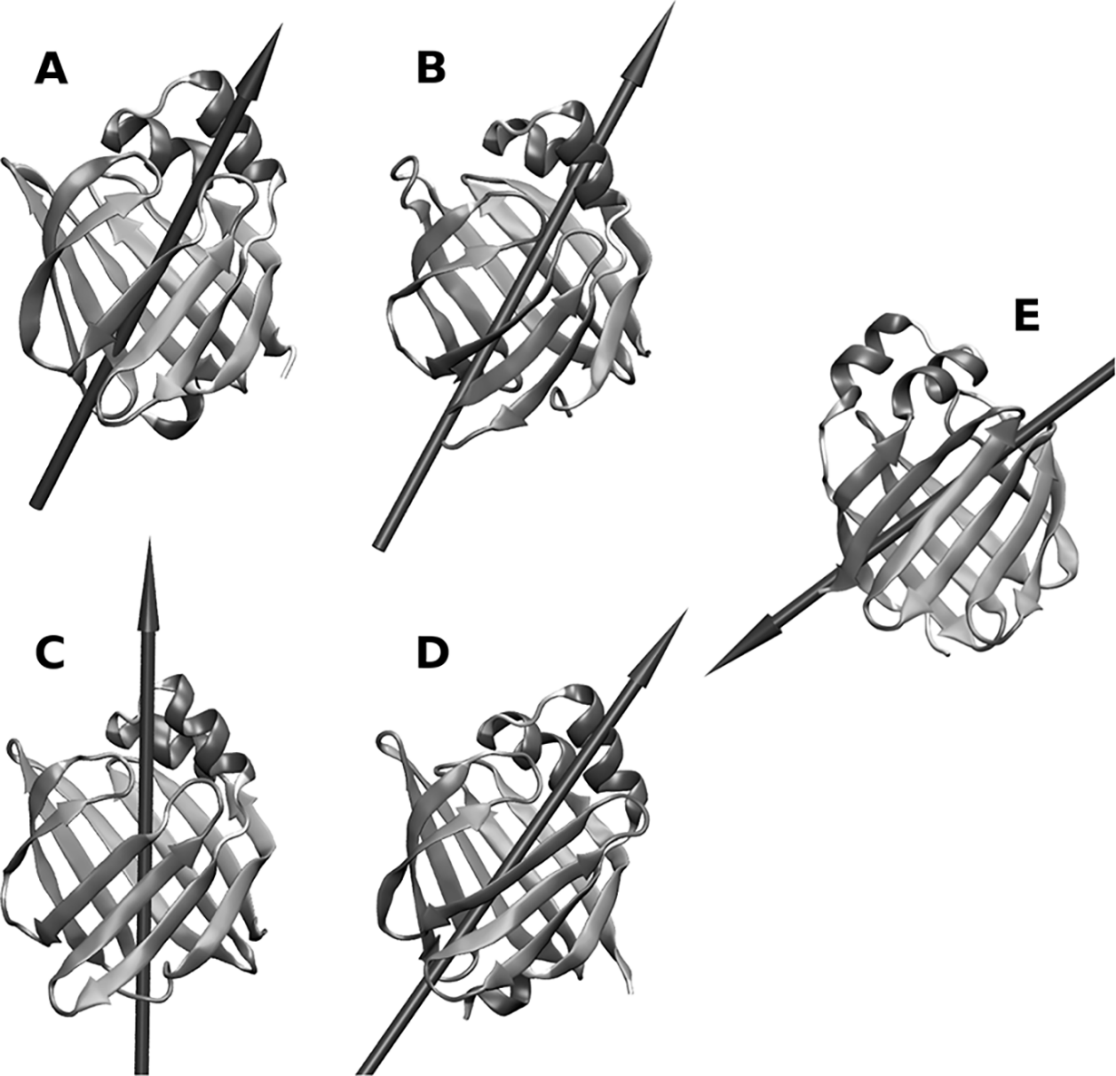
Cartoon representation of several human FABPs with their macrodipole. Macrodipoles (*μ*) are represented as arrows. The macrodipole was calculated according to GROMOS96 partial charges. A: Heart FABP (PDB ID 1G5W); *μ* = 158 D. B: Intestine FABP (PDB ID 1KZW); *μ* = 199 D. C: Adipocyte FABP (PDB ID 3FR4); *μ* = 361 D. D: Epidermal FABP (PDB ID 1JJJ); *μ* = 272 D. E: Liver FABP (PDB ID 2F73); *μ* = 151 D. Moment dipole vector scaling is not linear with the dipole moment magnitude.

Both ReP1-NCXSQ and L-BABP bind electrostatically to cationic lipid membranes at pH = 6.8 even when they have a net positive and negative electric charge respectively. We consider two main contributions to explain the binding: the interaction of the protein net charge and the interaction of the protein macrodipole with the membrane electric field. In the regime of low net charge and large macrodipole (ReP1-NCXSQ and L-BABP at neutral pH) the macrodipole interaction with the membrane electrostatic field is predominant and both proteins bind to charged membranes irrespective of their net charge and of the membrane polarity. In contrast, when the net charge is high and the macrodipole is small (L-BABP, low pH), the electrostatic repulsion between the positively charged protein and the cationic membrane is larger than the dipole-membrane atraction. Consequently, the binding decreased. This proposal is in agreement with the observation that both proteins acquired opposite orientations in anionic and cationic lipid membranes. This rationale can be generalized to many peripheral proteins that bind electrostatically to lipid membranes, such as proteinase 3 [49], phospholipase A2 [50] and ACBP [51]. We calculated the macrodipole for these proteins (not shown) and found that their predicted orientation is in agreement with the expected orientation of the macrodipole within the membrane electrostatic field. Particularly for FABPs, the dipole-field interactions are relevant for their biological function. FABPs transfer non-polar ligands to the lipid membrane by release and diffusion or by collision [52, 53]. Zamarreño et al. [14] showed that the energy profile for the collisional human IFABP as a function of geometrical coordinates has a minimum, while the diffusional rat LFABP displays a flat landscape. This suggests that a defined orientation is required for the collisional mechanism. We computed the macrodipole for several FABPs (Fig 8). These proteins deliver the ligand by the collisional mechanism (Fig 8A, Fig 8B and Fig 8C). They have a net, large dipole along the barrel, with the positive end pointing to the helix domain. The difference with the diffusional FABP (Fig 8E) is striking: the dipole points to the opposite end or is largely biased from the main axis. This analysis supports the hypothesis that the electrostatic interaction of the protein macrodipole with interfacial electric field drives the association of peripheral proteins to charged lipid membranes and is a major contribution to the protein orientation within the interface.

## Supporting information

S1 Fig The structure of EDMPC. Numbering of the atoms correlates with S1 Table.
(JPG)

S2 Fig DSSP analysis of Rep1-NCXSQ along the different simulations. (PNG)

S1 Table The partial electric charges on the atoms of EDMPC. See the corresponding atoms in S1 Fig.
(DOCX)
