# Supplementary figures and images for: Relevance of the protein macrodipole in the membrane-binding process. Interactions of fatty-acid binding proteins with cationic lipid membranes
Source: PLoS One. 2018 Mar 8;13(3):e0194154. doi: 10.1371/journal.pone.0194154 (PMC5843346; doi:10.1371/journal.pone.0194154)

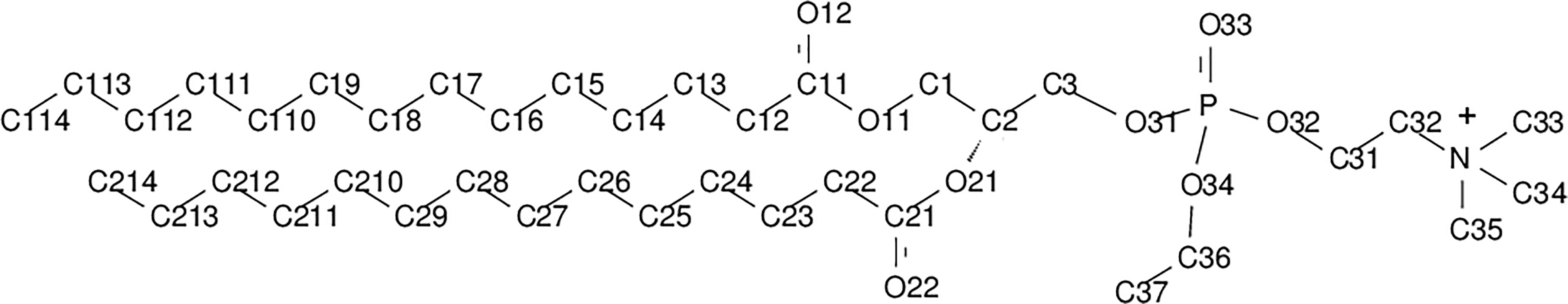

Supplement: S1 Fig — Numbering of the atoms correlates with S1 Table. (JPG) [file pone.0194154.s001.jpg]

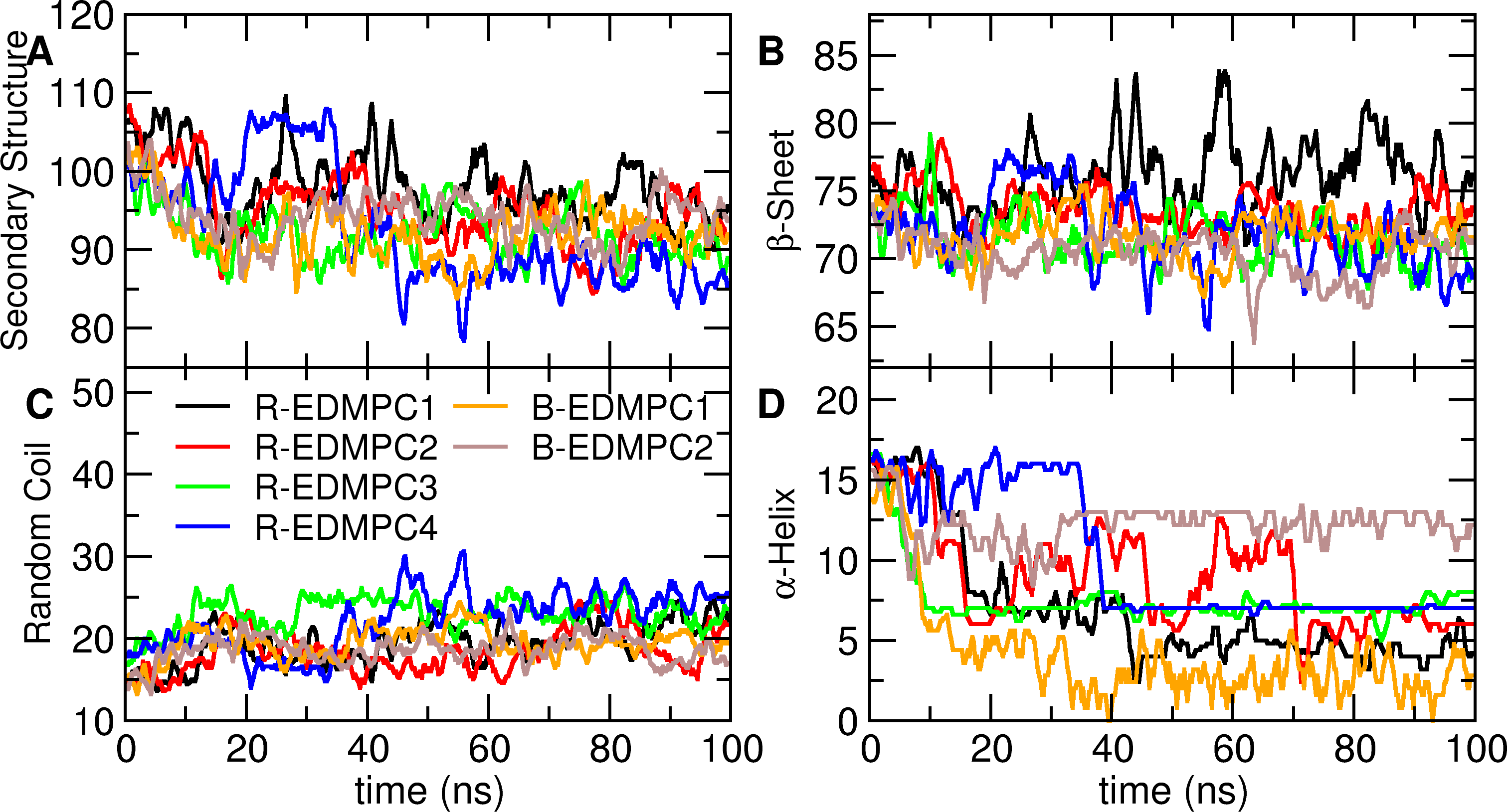

Supplement: S2 Fig — (PNG) [file pone.0194154.s002.png]
