# Supplementary material for: Relevance of the protein macrodipole in the membrane-binding process. Interactions of fatty-acid binding proteins with cationic lipid membranes
Source: PLoS One. 2018 Mar 8;13(3):e0194154. doi: 10.1371/journal.pone.0194154 (PMC5843346; doi:10.1371/journal.pone.0194154)

| **United Atom Name** | **Atom Type** | **Partial Charge** |
| --- | --- | --- |
| C36 | CH2 | 0.4 |
| C37 | CH3 | 0.5 |
| C33 | CH3 | 0.4 |
| C34 | CH3 | 0.4 |
| C35 | CH3 | 0.4 |
| N | NL | -0.5 |
| C32 | CH2 | 0.3 |
| C31 | CH2 | 0.4 |
| O32 | OA | -0.8 |
| P | P | 1.7 |
| O33 | OM | -0.8 |
| O34 | OA | -0.7 |
| O31 | OA | -0.7 |
| C3 | CH2 | 0.4 |
| C2 | CH1 | 0.3 |
| O21 | OE | -0.7 |
| C21 | CH0 | 0.7 |
| O22 | O | -0.7 |
| C22 | CH2 | 0.0 |
| C23 | CH2 | 0.0 |
| C24 | CH2 | 0.0 |
| C25 | CH2 | 0.0 |
| C26 | CH2 | 0.0 |
| C27 | CH2 | 0.0 |
| C28 | CH2 | 0.0 |
| C29 | CH2 | 0.0 |
| C210 | CH2 | 0.0 |
| C211 | CH2 | 0.0 |
| C212 | CH2 | 0.0 |
| C213 | CH2 | 0.0 |
| C214 | CH3 | 0.0 |
| C1 | CH2 | 0.5 |
| O11 | OE | -0.7 |
| C11 | CH0 | 0.8 |
| O12 | O | -0.6 |
| C12 | CH2 | 0.0 |
| C13 | CH2 | 0.0 |
| C14 | CH2 | 0.0 |
| C15 | CH2 | 0.0 |
| C16 | CH2 | 0.0 |
| C17 | CH2 | 0.0 |
| C18 | CH2 | 0.0 |
| C19 | CH2 | 0.0 |
| C110 | CH2 | 0.0 |
| C111 | CH2 | 0.0 |
| C112 | CH2 | 0.0 |
| C113 | CH2 | 0.0 |
| C114 | CH3 | 0.0 |


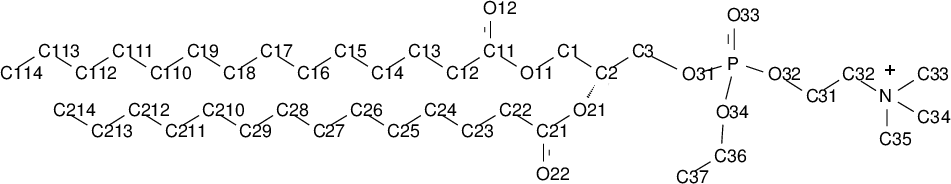

Supplement: S1 Table — See the corresponding atoms in S1 Fig. (DOCX) [file pone.0194154.s003.docx]
